# A Fusion Method of Gabor Wavelet Transform and Unsupervised Clustering Algorithms for Tissue Edge Detection

**DOI:** 10.1155/2014/964870

**Published:** 2014-03-23

**Authors:** Burhan Ergen

**Affiliations:** Department of Computer Engineering, Faculty of Engineering, Firat University, 23119 Elazig, Turkey

## Abstract

This paper proposes two edge detection methods for medical images by integrating the advantages of Gabor wavelet transform (GWT) and unsupervised clustering algorithms. The GWT is used to enhance the edge information in an image while suppressing noise. Following this, the *k*-means and Fuzzy *c*-means (FCM) clustering algorithms are used to convert a gray level image into a binary image. The proposed methods are tested using medical images obtained through Computed Tomography (CT) and Magnetic Resonance Imaging (MRI) devices, and a phantom image. The results prove that the proposed methods are successful for edge detection, even in noisy cases.

## 1. Introduction

Magnetic Resonance Imaging (MRI) and Computed Tomography (CT) devices are currently the most important diagnostic tools for medical examination. These imaging techniques provide a wealth of information about biological tissues and the condition of anatomical structures. The determination of tissue boundaries plays an important role in many medical applications aimed at identifying the abnormalities in anatomical structures and tissues [1–3]. Also, clinicians planning invasive or noninvasive treatments, such as surgery or radiotherapy, require the correct edge of tumours or tissues to be identified in order to operate. To determine the accurate edge of abnormal tissues helps clinicians to create or modify a treatment plan or to select the region and the path of the operation. Generally, the edge detection and the segmentation are performed by trained radiologists manually. Because the edge detection process is a time consuming task and is subject to radiologist error, researchers have concentrated on developing edge detection methods which detect the accurate edge [4–6]. The detection of the accurate or the acceptable edge is very difficult due to the properties of the analysed image and the variety of modalities. The medical images received from medical devices, such as MRI or CT, are always corrupted by noise and the artifacts of the devices [7–10]. Therefore, one of the difficulties for edge detection of medical images is the effect of noise and artifacts disturbing the edge of the analyzed images.

It is not enough that detected edges are visually soft and nice. The detected edges should be determined accurately. In the edge detection process, it is commonly considered that an edge detection method should detect all the edges of the objects in the examined image at their correct positions and should not detect non-edge. In an image, the edge is generally obtained using gradient, texture, and intensity, which are the measurable features of the examined image. Early edge detection methods employ local operators to compute the first and second gray-level gradients of an image in the spatial domain. After this, the local maximum locations of the first derivatives or the second derivatives are assumed as the edge points in the analyzed image. In the same way that classical edge detection operators considered benchmark methods [11], Sobel operator, Prewitt operator, LOG operator, and Robert operators compute the derivatives or the gradients. Some edge-detector methods use second-order derivatives like Laplacian or DoG (Difference of Gaussian). If the image to be analyzed has poor contrast values and noise between the interested regions and the weak boundaries, the operator-based edge detection methods encounter difficulties in the detection of the correct edge [7, 8].

However a medical image cannot be free of noise and artifacts; the medical images acquired from CT or MRI devices usually suffer from noise and artifacts. These factors reduce the quality of the medical images used to detect the edges. Therefore, a lot of edge detection methods have been put forward recently which detect the boundaries of tissues in a medical image such as wavelet transform, mathematical morphological method, neural networks, or fuzzy methods [12–15].

Even if these studies are successful in identifying the external shape of the interested tissues, it is observed that the usage of the prior information and the shape models does not accurately identify the internal structural changes of the interested tissues. In order to identify the external and internal edge of a tissue, automatic edge detection methods could help because a shape model is not able to model the edge of the internal structures. Thus, some new methods based on soft computing algorithms such as fuzzy, neural, or genetic algorithms have been proposed so that the internal structures may be appropriately determined [16, 17].

Since automatic edge detection and segmentation are very difficult, some studies concentrating on a particular problem have used shape models or prior information about the tissues [18]. Although soft computing algorithms have been successful in providing more reliable edges than the traditional and shape modeling methods, it is also reported that these methods are highly sensitive to noise and artifacts [11, 19, 20]. FCM and *k*-means segmentation methods are frequently encountered methods among the soft computing methods used for segmentation problems [21, 22].

Whereas these methods just consider the intensity of the given image, it is reported that FCM cannot give good results if the image is noisy or does not have homogeneous structures [13, 19, 23]. If the existence of noise and artifacts in medical images is taken into consideration, it is clear the FCM is insufficient for the detection of accurate edge. However many algorithms have been proposed for the improvement of FCM; the FCM-based algorithms, when used alone, are still not robust against noise and nonhomogeneity [19, 21, 24]. The performances of soft computing methods for edge detection are decreased when they are used alone because the noise obscures the weak edges. Traditional edge detectors and soft computing algorithms, which may identify the edge of the internal structures for tissue segmentation, are highly sensitive to noise. If the image has a low signal-to-noise ratio, the traditional edge detectors and the soft computing algorithms fail to determine the contours of the anatomical structures correctly [25].

Therefore, many noise suppression methods have been proposed for the enhancement of the image to be analyzed. However some of the image denoising methods use the pixel relation in spatial domain; the rest assumes that the rapid change in frequency domain refers to noise [26].

While several noise suppression methods which filter the background noise of an image have been proposed, the traditional noise reduction methods are based on the median filter such as Adaptive Weighted Median Filter (AWMF) or the mean filter such as Homogeneous Region Growing Mean Filter (HRGMF) [27]. An improved version of the HRGMF filter, Aggressive Region Growing Filter (ARGF), is proposed in [28, 29]. This filter uses an adaptive homogeneity threshold instead of the constant threshold value of the HRGMF filter.

Some of the image denoising methods work on frequency domain such as wavelet-based methods. In wavelet-based image denosing process, the image is decomposed into four subimages with respect to their frequency bands in one level decomposition. Afterwards, the small detail coefficients are properly eliminated [26]. Any noise reduction method makes the image blurred less or more. However the noise reduction methods increase the SNR of the given image, and the weak edges in the images become invisible and undetected.

**Figure 1:**
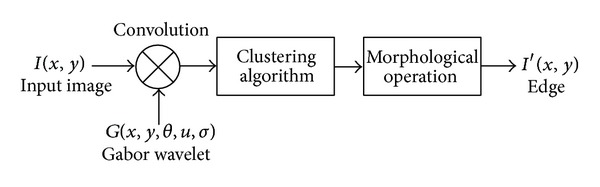
The proposed edge detection method.

Although edge detection is a very difficult task, humans can easily determine the boundaries within an image without realizing consciously that they are doing so. The human visual system can be modeled as a filter bank. This filter bank can be represented using Gabor functions having different orientation and frequencies. The output of the representation using Gabor function can be accepted as the responses of the human visual system. In particular, the Gabor wavelet transform has demonstrated good performance in texture representation and discrimination [30, 31], and it has been successfully applied to face recognition, object detection [32], palm print recognition [33], and also object tracking [34]. Therefore, we have developed a technique integrating the advantages of Gabor wavelet transform and unsupervised clustering algorithms, FCM and *k*-means. The GWT is used as a tool for enhancing the edge of images while suppressing noise. The clustering algorithms convert the gray level edge image into a binary image without any thresholding process. The estimation of an appropriate threshold value is a very difficult task if the histogram of the given image has multiple valleys. Here, we have used the clustering methods, *k*-means and FCM, for the binary conversion avoiding any thresholding process.  Figure 1 demonstrates the proposed edge detection method. The proposed algorithm has three steps: the convolution of the input image with Gabor functions, a clustering algorithm to obtain the binary image, and morphological operation to detect the edge.

The paper is organized as follows.  Section 2 introduces GWT. In Section 3, *k*-means and the FCM, the most known and used unsupervised algorithms, are presented. In Section 4, the experiments are performed on medical images and a phantom image, in comparison with traditional edge detection methods, Prewitt, Canny, and Sobel. The conclusion is given in Section 5.

## 2. Gabor Wavelet Transform (GWT)

Gabor functions, with different frequencies and orientation, can model the human visual system as a filter bank [33, 35]. A Gabor wavelet can be described as a Gaussian kernel function modulated by a sinusoidal plane wave that has an optimal location in both the frequency domain and the space domain. In literature, there is a significant amount of computer vision applications using Gabor functions, such as texture segmentation, image analysis, and discriminations [33, 36].

Gabor wavelets reveal the directional features of an image while providing a fine adjustment for frequency properties [31, 36, 37]. The capability of frequency adjustment is particularly important for the reduction of the background noise in medical images. The preservation of the features of the edge is the most important thing in the noise reduction process. The 2D Gabor wavelet is defined as follows:

(1) G(x,y,θ,u,σ)=12πσ2exp⁡{−x2+y22σ2}exp⁡{2πj(uxcos⁡θ+uysinθ)},

where *u* is the frequency of the sinusoidal wave, *θ* adjusts the orientation of the wave, *σ* is the standard deviation of the Gaussian function in the *x* and *y* direction, and $j=\sqrt{-1}$. The output of the Gabor filtering can be given as a 2D convolution of the input image *I*(*x*, *y*) and *G*(*x*, *y*) for particular *u*, *θ*, and *σ*. The result is a 2D complex signal because Gabor wavelet is complex. The absolute of this signal is an image preserving the features of the edge. When the wave vector is perpendicular to the edge, the Gabor wavelets enhance the edge and remove the background information. The result image of the convolution demonstrates the local properties indicating the edge of the analyzed image [31]. Kernels related to angles are obtained by setting orientation factor.

**Figure 2:**
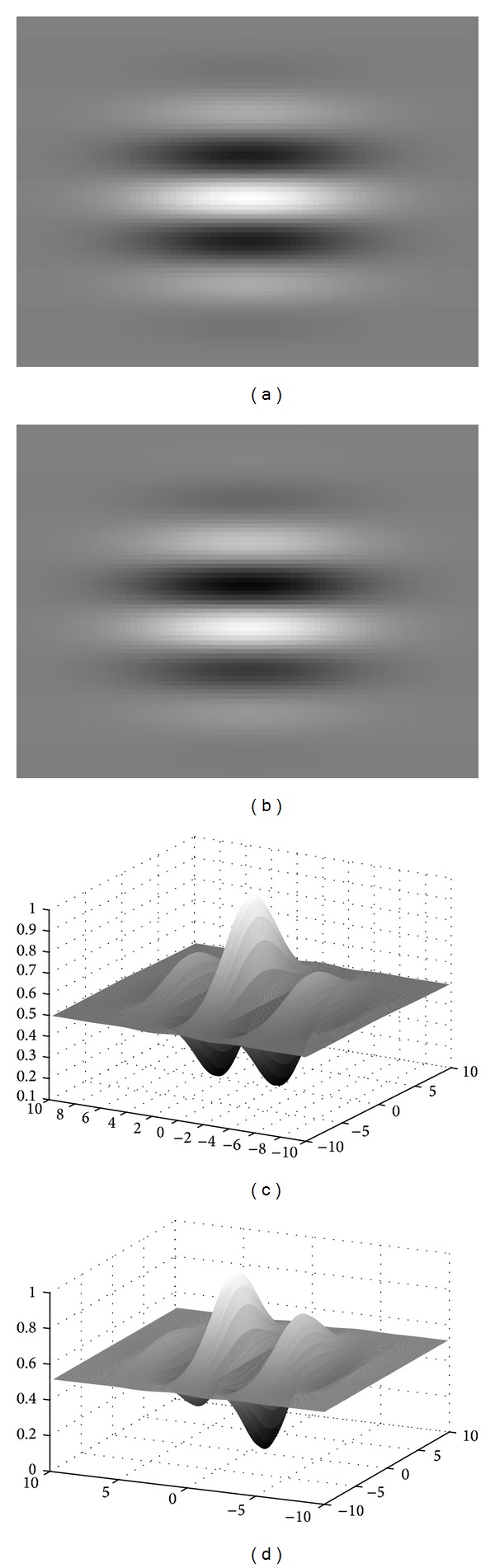
A two-dimensional Gabor wavelet; ((a), (c)) real part, ((b), (d)) imaginary part.

Figure 2 shows a different representation for the 2D Gabor wavelet *G*(*x*, *y*) with parameters *σ* = 0.03, *u* = 0.3 and orientation factor *θ* = 0. Figures 2(a) and 2(c) represent the real part, and Figures 2(b) and 2(d) represent the imaginary part. Actually, Gabor wavelet is a complex wavelet with a few important oscillations relating to frequency parameter. The magnitude decay rate of the oscillations depends on the value of *σ*. The characteristics of 2D Gabor wavelet are particularly appropriate to extract the directional features, and the waveform is suitable to preserve the edge pixels while suppressing the noise, which is encountered in medical images.

## 3. Unsupervised Clustering

### 3.1. *K*-Means Clustering

A typical unsupervised clustering algorithm is *k*-means which is attractive in practice because it is simple and fast [38, 39]. The algorithm tries to partition the given input data into *k* disjoint clusters *c*_*i*_. For this purpose, it searches the cluster centers by minimizing the sum of squared distances of each data point (*x*_1_, *x*_2_,…, *x*_*N*_) to its nearest cluster centre *c*_*j*_. The measure of the distance is commonly chosen as Euclidian distance to minimize the following mean square error (MSE) cost function:

(2) $$\begin{array}{c} C_{\text{MSE}}=\sum_{i=1}^{k}\sum_{x_{j}\in c_{i}}^{}{||x_{j}-c_{i}||}^{2}, \end{array}$$

where *C*_MSE_ shows the cost of an examined pixel to assign a cluster, which is the distance.

*x* and *c* are the data point and the cluster centre. It can be said that *x* is in cluster if ||*x*_*j*_ − *c*_*i*_|| is the minimum of all the *k* distances.

*K*-means algorithm can be summarized as follows.

**Step 1**

Initializations of centre location (*c*_1_, *c*_1_,…, *c*_*k*_).

**Step 2**

Assigning each *x*_*i*_ to its nearest cluster centre *c*_*k*_.

**Step 3**

Deciding the membership of each pixel to the *k* clusters, whose centroid is closest to that pixel.

**Step 4**

Setting *c*_*i*_ to be the centre of mass of all points in cluster *C*_*i*_ for all *k* cluster centers.

### 3.2. Fuzzy* C*-Means Clustering

The clustering process can be expressed as grouping pixels according to the similarities of their features. A clustering algorithm can provide a way of differentiating the regions in an image. Several methods based on the ideas using clustering algorithms have been proposed to partition an image into regions. As an unsupervised technique, Fuzzy *c*-means (FCM) clustering, was proposed by Bezdek et al. [40] as one of the widely used techniques to determine the boundaries of the objects in an image. It is considered that the reason for the high performance of FCM is due to the fact that through this process each pixel is assigned to a cluster or segment. FCM algorithm groups similar pixels according to their features because an image is represented by its features, such as histogram properties [41].

The cost function, which depends on the distance between the cluster centers and pixels, is calculated iteratively to find a minimum value. The FCM determines the clusters when the cost is minimized. The studies using FCM-based segmentation algorithms reported that FCM-based segmentation methods preserve more information than crisp and hard segmentation methods [42]. However, one of the drawbacks of FCM-based segmentation is sensitivity to noise and imaging artifacts, which is frequently encountered in medical imaging. This disadvantage is due to that fact that spatial information is not taken into account. In FCM, the cluster centers are repositioned after the calculation of an objective function used as *c*-means. There is flexibility in FCM because the objective function includes a membership value to a cluster [24, 41].

In FCM algorithms, each of the pixels is assigned to suitable categories by using a membership function after calculation of a cost function. The cost function is calculated iteratively to find the minimum value using Euclidean distance between the examined pixel and the centre to be assigned. This can be formulated as follows:

(3) $$\begin{array}{c} E=\sum_{j=1}^{N}\sum_{i=1}^{C}\mu_{ij}^{k}||p_{j}-c_{i}||, \end{array}$$

where *E* shows the cost of an examined pixel to assign a cluster. *μ*_*ij*_ and *c*_*i*_ represent a membership of a pixel to a cluster and the cluster centre, respectively. ||·|| denotes absolute value operator. *k* is used to adjust the fuzziness as a constant value.

Here, the membership can be expressed as the probability of a pixel belonging to a cluster. This probability depends on the distance of the pixel to a cluster centre. The probability of the pixel belonging to a cluster can be calculated as follows:

(4) $$\begin{array}{c} c_{i}=\frac{\sum_{j=1}^{N}\mu_{ij}^{k}x_{j}}{\sum_{j=1}^{N}\mu_{ij}^{k}}, \end{array}$$

(5) $$\begin{array}{c} \mu_{ij}={\left({\sum_{m=1}^{C}\left(\frac{||x_{j}-c_{i}||}{||x_{j}-c_{m}||}\right)}^{2/(k-1)}\right)}^{-1}. \end{array}$$

The FCM algorithm process these two equations iteratively.

## 4. Experiments

**Figure 3:**
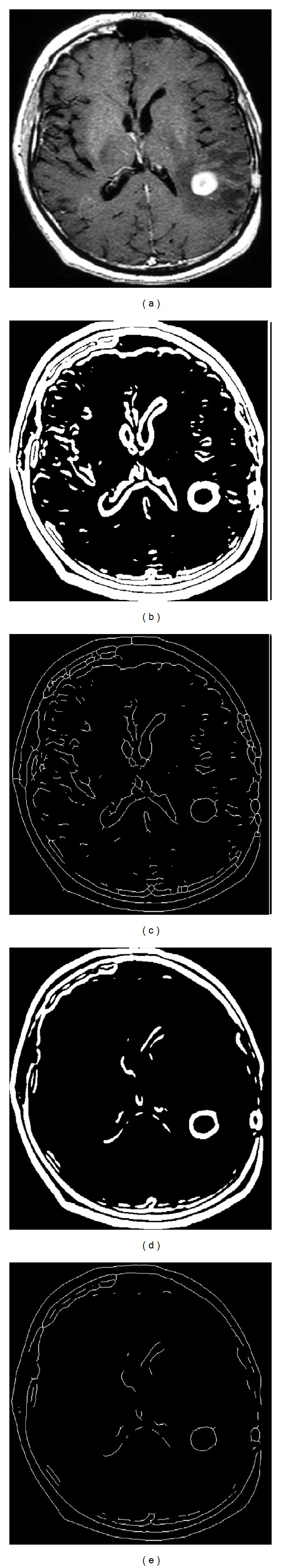
Edges using *k*-means and FCM clustering after GWT; (a) original, (b) *k*-means clustering, (c) skeleton of (b), (d) FCM clustering, and (e) skeleton of (d).

We have tested our method on several medical images acquired from CT and MRI imaging devices. The edges of the CT brain scan and abdominal images are determined using GWT, an unsupervised clustering algorithm, and morphological skeletonisation.  Figure 3 represents a CT brain scan image including a tumour, clustering results, and the tissue edges. The edges are obtained in the three steps in our approach. After applying Gabor wavelet transformation to find out directional edge information, an unsupervised clustering algorithm is used to convert the gray level image into a binary image, which still contains irrelevant pixels. Then, some morphological operations are used to remove the irrelevant pixels in the binary image. As clustering algorithms, we have favored *k*-means and FCM clustering algorithms because they are unsupervised methods. As a morphological method, the skeletonisation is used in order to eliminate the irrelevant pixels in the binary image.

**Figure 4:**
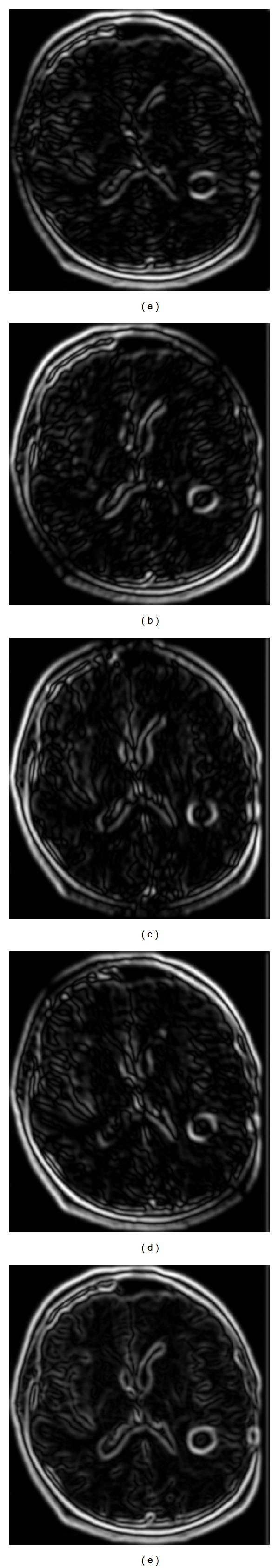
The GWT of a CT scan brain image for different orientation factors (*σ* = 0.1 and *ω* = 0.005); (a) *π*/4, (b) *π*/2, (c) 3*π*/4, (d) *π*, and (e) total result.

**Figure 5:**
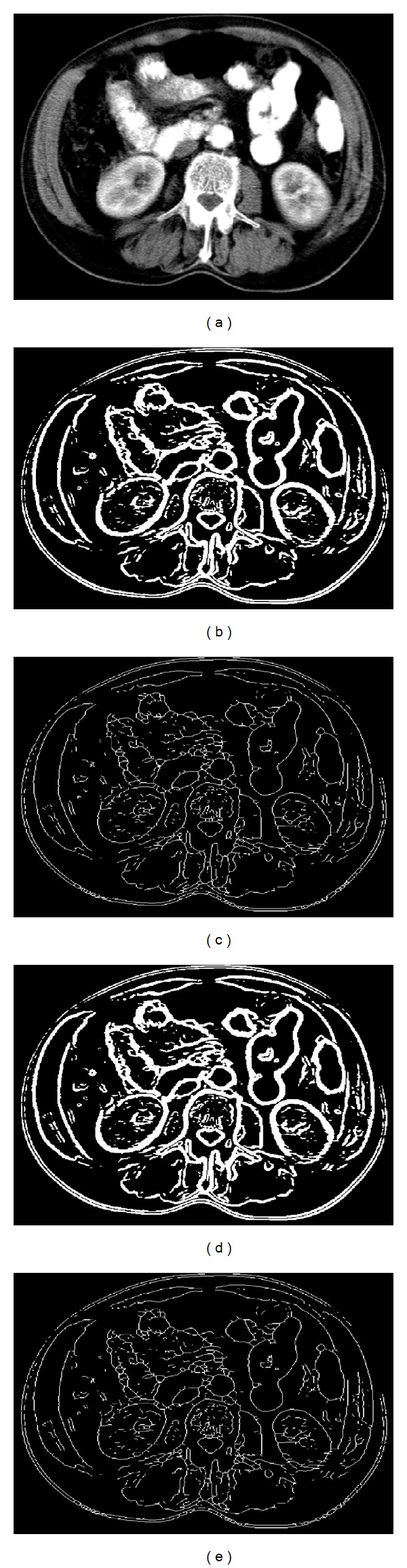
Edges of an abdominal CT image (*σ* = 0.18 and *ω* = 0.0005); (a) original, (b) *k*-means, (c) skeleton of (b), (d) FCM, (e) skeleton of (d).

Figure 4 represents the GWT results of the brain image given in Figure 3 using four different orientations (*π*/4, *π*/2, 3*π*/4, and *π*). This figure shows how the orientation of GWT plays an important role for the enhancement of the boundaries. The image in Figure 4(e) is the total resulting image of the GWT, which contains the total edge information. An unsupervised clustering method is used to convert the gray level image obtained as GWT result into a binary image.  Figure 5 represents the binary image obtained using unsupervised methods and their skeletons as an example of abdominal images.

**Figure 6:**
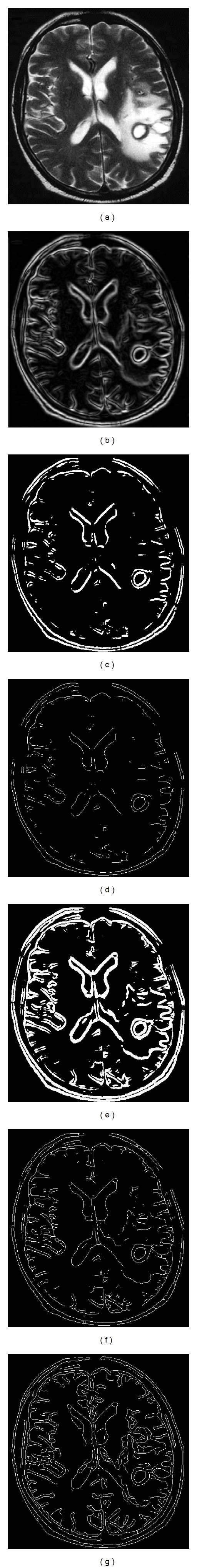
Edges of a CT scan brain image (*σ* = 0.3 and *ω* = 0.05); (a) original, (b) GWT, (c) *k*-means, (d) skeleton of (c), (e) FCM, (f) skeleton of (e), and (g) Canny.

**Figure 7:**
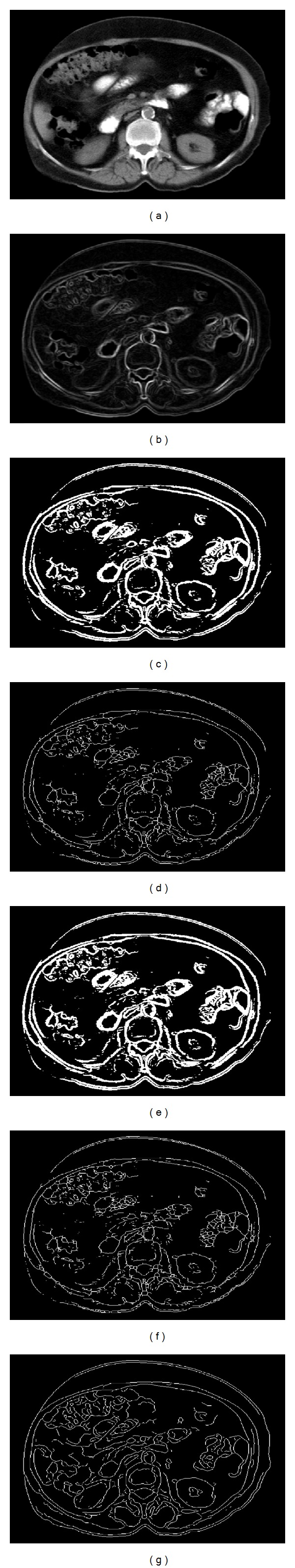
Edges of a CT scan abdominal image (*σ* = 0.18 and *ω* = 0.0005); (a) original, (b) GWT, (c) *k*-means, (d) skeleton of (c), (d) FCM, (e) skeleton of (d), and (g) Canny.

Figures 6 and 7 also represent the results of the proposed method for a CT brain scan image and an abdominal image, respectively. Because the Canny edge detection method is widely used to present the ground truth images in many applications [43], the edge detection results using the Canny method are also given in these figures to carry out a visual comparison.

In fact, it is never possible to identify the accurate edge of a real image. Although there is not a reliable methodology to put forward an appropriate ground truth edge [44], the edge of synthetic image and the manually drawn edge of a real image are used as the ground truth edge.

Another difficulty is how to define the quality parameter in order to estimate the integrity of edge detection because of application dependency. While several methods have been proposed in the literature to measure the performance of an edge detector objectively, there is no agreement on the quality parameter about edge detection. Nevertheless, the misclassification rate (MCR) and Pratt's figure of Merit (FOM) are proposed to measure the similarity between the ground truth edge and the detected edge in literature.

The MCR can be defined as follows:

(6) $$\begin{array}{c} \text{MCR}=\frac{\sum \left|B_{A}\cap B_{D}\right|+\sum \left|F_{A}\cap F_{D}\right|}{\sum \left(B_{A}+F_{D}\right)}\times 100\%, \end{array}$$

where *F*_*A*_ and *F*_*D*_ refer to the foreground pixels of actual and detected image while *B*_*A*_ and *B*_*D*_ refer to the background pixels of the actual and the detected image. Indeed, the operation of the numerator refers to logical “exor” operation [22, 24]. The less value of MCR indicates that a good detection is done.

The definition of FOM can be given as follows:

(7) $$\begin{array}{c} \text{FOM}=\frac{1}{\max\left(N_{t},N_{d}\right)}\sum_{i=1}^{N_{d}}\frac{1}{1+\alpha L{\left(i\right)}^{2}}, \end{array}$$

where *N* refers to the number of edge pixels, and subscripts *d* and *t* denote the detected edge and the accurate edge, respectively. *L*(*i*) shows the distance between the *i*th accurate edge pixel and the detected edge pixel. And parameter *α* is generally accepted as 1/9 as a scaling factor [43]. When an accurate edge is not detected, or a false edge is detected, or the detected edge is far from the accurate edge, FOM value increases. If the edge is perfectly detected, FOM value is 1. Otherwise, it may decrease to zero. The difference between the comparison methods, MCR and FOM, can be expressed as follows. Whereas MCR algorithms accept only the exact overlapped pixel points between the estimated edge and the accurate edge, the FOM algorithms accept the pixel points of the founded edge if they are very near to the accurate edges.

**Figure 8:**
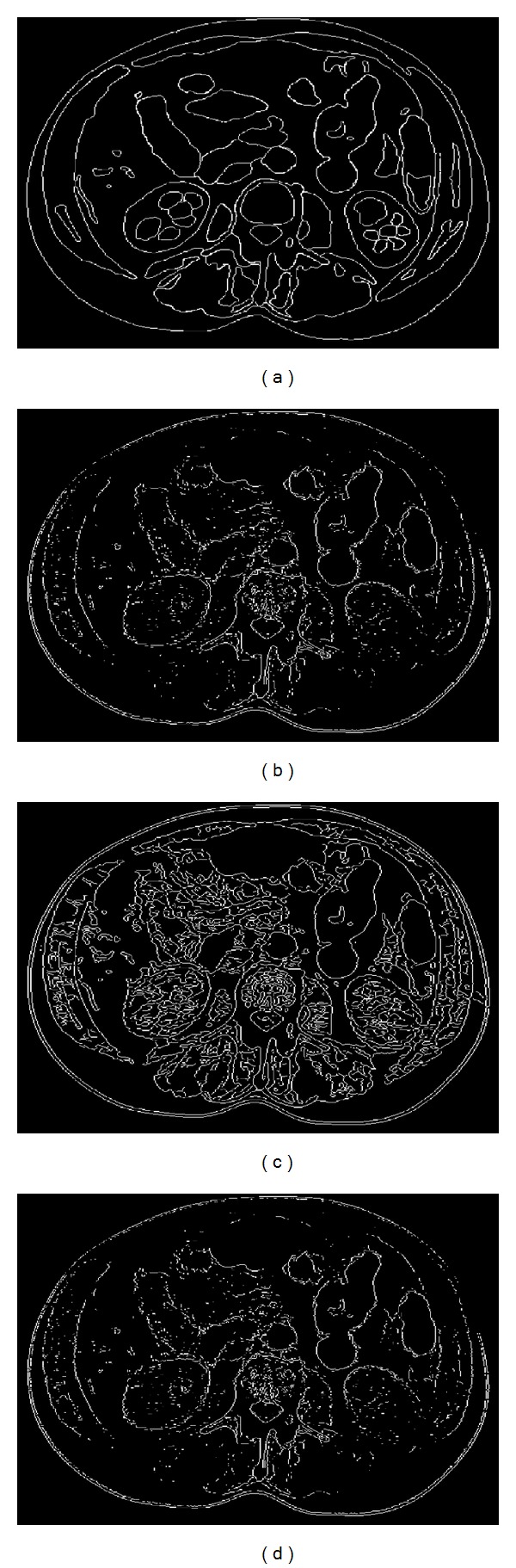
Edges of Figure 5(a); (a) manually drawn ground truth edge, (b) Prewitt, (c) Canny, and (d) Sobel.

To present the performance of our proposed method, we applied both of the methodologies on a synthetic phantom image and a real image to obtain the appropriate ground truth edges. The images are ordinary CT and MRI images, and no prefiltering procedure is applied. A real and manually annotated approach was performed under the supervision of a radiologist.  Figure 8(a) represents the manually ground truth edge of an abdominal image given as an example in Figure 5(a). The edges of the classical methods (Canny, Sobel, and Prewitt), which are accepted as benchmark methods in literature, are also given in Figure 8. Because many edge detection methods are proposed in the literature, we just compared to the most know methods accepted as benchmark.

To evaluate the performance of the proposed method, the MCR and FOM values were calculated using the edge given in Figure 8(a) and other methods, *k*-means, FCM, Prewitt, Canny, and Sobel.  Table 1 represents the result of the comparison. These results prove that the performance of the proposed method is higher than the other methods. In particular, FCM represents the highest performance with 0.7981 of FOM values. The Canny edge detection method, which is widely used to obtain a ground truth edge, has the smallest FOM value in the table. The reason for this result is that medical images are always noisy images, and the Canny edge detection method is highly sensitive to noisy signals. *K*-means-based edge detection also has higher FOM value than Canny, Prewitt, and Sobel methods. According to MCR values in Table 1, FCM- and *k*-means-based GWT edge detection methods have smaller values, 4.8653 and 5.6980, respectively. The fact that MCR results are compatible with FOM results supports that GWT-based edge detection methods have higher performances.

**Figure 9:**
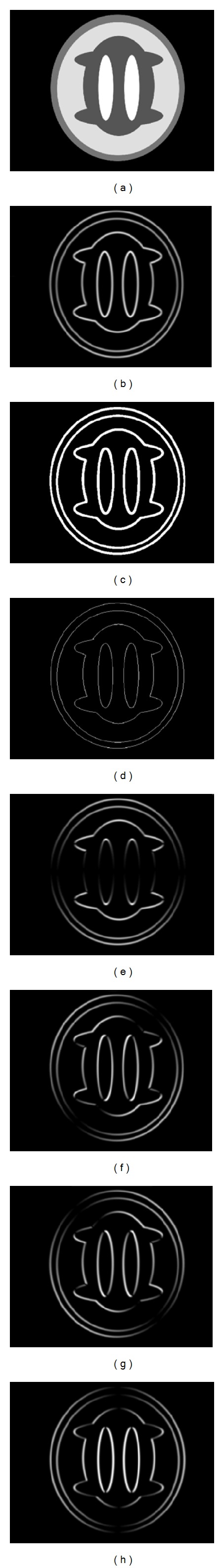
The simulated image and its GWT (*σ* = 0.24 and *ω* = 0.0055); (a) original phantom image, (b) GWT, (c) *k*-means, (d) skeleton of (c), (e) *π*/4, (f) *π*/2, (g) 3*π*/4, and (h) *π*.

As stated, it is very difficult to find the edge of a medical image corrupted by noisy signals. Medical images are generally low-density and noisy images depending on the type of imaging device. Furthermore, no medical imaging device can operate independently of the noise. Therefore, the resistance of an edge detection method to noise should be taken into consideration, particularly in medical imaging. In order to measure the resistance to noisy signals, we have performed another experiment using a synthetic phantom image given in Figure 9(a). Why we use the phantom image and its noisy form is to make a more objective assessment.

The phantom image is constituted using several overlapping ellipses. It is assumed each ellipse indicates to a tissue. The accurate edge accepted as the ground truth edge was determined when the phantom image was constituted. The MCR and FOM values are computed for the phantom image to conclude an evaluation in order to measure the performance of edge detection methods on the phantom image. The results given in Table 2 show that the proposed algorithm can detect the edges more precisely than the classical method even in the presence of noise. It can be considered that better results have been yielded by the proposed method because the GWT acts as a gradient operator while suppressing noise. The last row (∗) shows the results of the edge detection methods after applying a median filter on the noisy phantom image. The filter operation is applied to the noisy phantom image only when using classical methods. No filter operation is applied to the phantom image when using the GWT-based methods. Even in this case, the proposed methods give higher FOM values and smaller MCR values, as seen in Table 2. This last result proves that GWT is successful even in noisy cases.

The FOM values of the proposed method may change slightly with respect to the trail number because *k*-means and FCM clustering methods choose the centroid arbitrarily. According to the results of the phantom image, it is observed that the FCM clustering algorithm is more sensitive than the *k*-means clustering algorithm. Nonetheless, the proposed method using *k*-means and FCM is very successful compared to other methods.

## 5. Conclusion

This work presents two methods of edge detection based on the GWT. These two proposed methods use *k*-means and FCM clustering method to convert a gray level image into a binary image. The main idea of the proposed method is to integrate the information obtained from the GWT at a different orientation and to incorporate the use of a clustering method. The effect of the GWT can be seen on the regional boundaries of the given image. The GWT enhances the edge information and suppresses the noisy signals in a given image. The tests prove that both methods have a great performance particularly in noisy conditions.

In this paper, we have proposed two kinds of edge detection methods based on GWT. Other methods using GWT could be conceptually explored and adapted. The results showed that the directional information from GWT provides a competitive advantage for edge analysis and detection. Since GWT uses three parameters, sigma, frequency, and orientation, it can be adapted for application-dependent images.

## Figures and Tables

**Table 1:** MCR and FOM results for the manually ground truth edge in Figure 8(a).

MCR results					FOM results				
*k*-means	FCM	Prewitt	Canny	Sobel	*k*-means	FCM	Prewitt	Canny	Sobel
5.6980	4.8653	6.1733	9.3037	6.2557	0.7195	0.7981	0.7010	0.5020	0.7094

**Table 2:** MCR and FOM results for the phantom image in Figure 9.

PSNR	MCR results					FOM results				
	*k*-means	FCM	Prewitt	Canny	Sobel	*k*-means	FCM	Prewitt	Canny	Sobel
35.01	0.9384	0.9285	0.8934	0.7954	0.8968	0.9710	0.9710	0.9723	0.9752	0.9722
30.01	0.9182	0.9350	0.9007	16.8981	0.8938	0.9712	0.9709	0.9721	0.1902	0.9723
25.02	0.9548	0.9563	0.9102	18.1576	0.9243	0.9700	0.9701	0.9614	0.0765	0.9622
23.00	1.2249	1.3115	2.9491	21.2528	2.9976	0.9429	0.8958	0.4788	0.0658	0.4690
23.00*	1.2196	1.3355	2.0824	7.6271	2.0962	0.9351	0.8985	0.7296	0.1831	0.7236

*Preprocessed using median filter.
